# Substrate selectivity and inhibition of histidine JmjC hydroxylases MINA53 and NO66†

**DOI:** 10.1039/d2cb00182a

**Published:** 2023-01-12

**Authors:** Vildan A. Türkmen, Jordi C. J. Hintzen, Anthony Tumber, Laust Moesgaard, Eidarus Salah, Jacob Kongsted, Christopher J. Schofield, Jasmin Mecinović

**Affiliations:** a Department of Physics, Chemistry and Pharmacy, University of Southern Denmark, Campusvej 55 5230 Odense Denmark mecinovic@sdu.dk; b Department of Chemistry and the Ineos Oxford Institute for Antimicrobial Research, Chemistry Research Laboratory, University of Oxford, 12 Mansfield Road OX1 3TA Oxford UK christopher.schofield@chem.ox.ac.uk

## Abstract

Non-haem Fe(ii) and 2-oxoglutarate (2OG) dependent oxygenases catalyse oxidation of multiple proteins in organisms ranging from bacteria to humans. We describe studies on the substrate selectivity and inhibition of the human ribosomal oxygenases (ROX) MINA53 and NO66, members of the JmjC 2OG oxygenase subfamily, which catalyse C-3 hydroxylation of histidine residues in Rpl27a and Rpl8, respectively. Assays with natural and unnatural histidine analogues incorporated into Rpl peptides provide evidence that MINA53 and NO66 have narrow substrate selectivities compared to some other human JmjC hydroxylases, including factor inhibiting HIF and JMJD6. Notably, the results of inhibition assays with Rpl peptides containing histidine analogues with acyclic side chains, including Asn, Gln and homoGln, suggest the activities of MINA53/NO66, and by implication related 2OG dependent protein hydroxylases/demethylases, might be regulated *in vivo* by competition with non-oxidised proteins/peptides. The inhibition results also provide avenues for development of inhibitors selective for MINA53 and NO66.

## Abstract 2

Ribosomal histidine hydroxylases MINA53 and NO66 exhibit narrow substrate selectivities for ribosomal protein L27a/L8 peptides possessing histidine analogues. Selected Rpl peptides display potent inhibition against MINA53 and NO66, providing a basis for inhibitor design.

## Graphical abstract

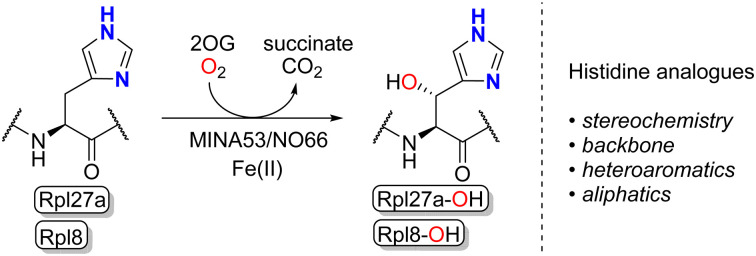

## Introduction

2-Oxoglutarate (2OG) dependent oxygenases catalyse the oxidation of multiple substrate types in organisms ranging from bacteria to humans.^1,2^ Reactions catalysed by them include hydroxylations, desaturations, halogenations, and oxidative ring forming processes.^1^ Early work concerning the substrate and product selectivities of 2OG oxygenases and the structurally related oxidase isopenicillin N synthase revealed that they have potential to be promiscuous, both in terms of the substrates they accept and the types of reactions they catalyse.^2^ In humans 2OG oxygenases play roles in processes including the regulation of transcription, collagen biosynthesis, lipid metabolism, and the hypoxic response.^3^ 2OG oxygenases have also been shown to catalyse the hydroxylation of ribosomal proteins.^4,5^

**Fig. 1:**
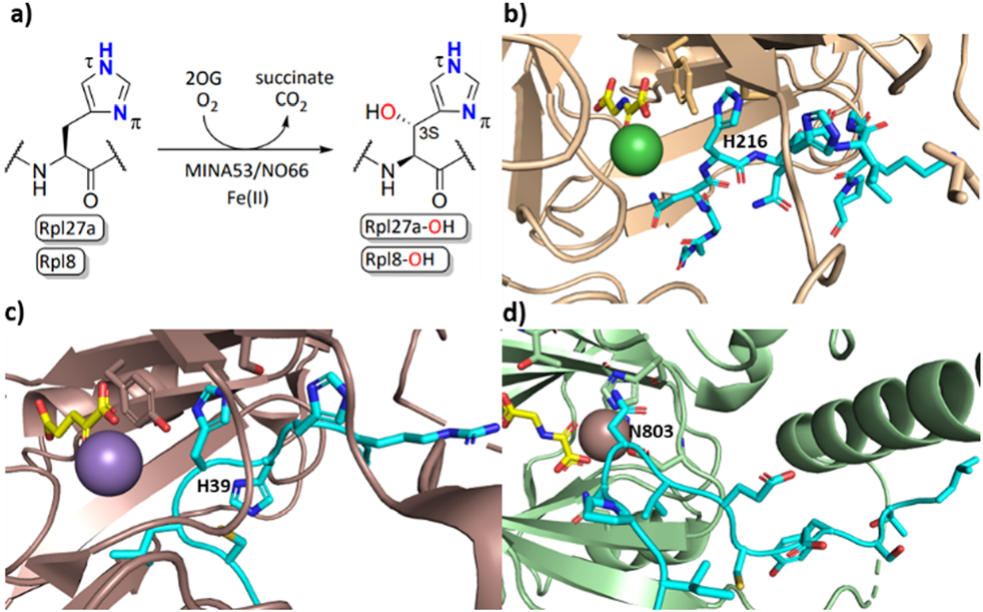
MINA53/NO66-catalysed hydroxylation of histidine residues in ribosomal proteins. (a) MINA53/NO66-catalysed β-hydroxylation of Rpl27a-His39 and Rpl8-His216, respectively; (b) view from a crystal structure of NO66 (wheat) complexed with a Rpl8-His216 fragment peptide (cyan), Ni (green, substituting for catalytically active Fe(ii)) and NOG (*N*-oxalylglycine, a 2OG mimic inhibitor, yellow); (PDB ID: 4Y3O); (c) view from a crystal structure of MINA53 (dark salmon) complexed with a Rpl27a-His39 fragment peptide (cyan), Mn (purple, substituting for Fe(ii)) and 2OG (yellow); (PDB ID: 4BXF); (d) view from a crystal structure of FIH (green) in complex with a HIF-1α-Asn803 containing fragment peptide (cyan), Fe and NOG (yellow); (PDB ID:1H2K).

The human ribosomal oxygenases (ROX) MINA53 (9 MYC-induced nuclear antigen 53) and NO66 (Nucleolar protein 66) belong to the JmjC subfamily of 2OG oxygenases.^5,6^ In bacteria, YcfD, which is structurally homologous to MINA53/NO66, catalyses C-3 hydroxylation of Arg81 of the ribosomal protein L16 (Rpl16).^5,7,8^ Human MINA53 and NO66, however, catalyse (3*S*)-hydroxylation of histidine residues in the ribosomal proteins Rpl27a or Rpl8, respectively (Fig. 1a–c). MINA53 and NO66 are also reported to have histone lysine demethylase (KDM) activity, *i.e.*, on H3K9 for MINA53 and on H3K4/K36 for NO66.^9,10^ The genes encoding for both MINA53 and NO66 are highly expressed in several cancers with poor prognosis, suggesting that they may be of interest as cancer drug targets.^11–13^ However, the physiological roles of MINA53 and NO66 have not yet been fully defined.

Apparently reflecting work on microbial 2OG oxygenases, some human 2OG oxygenases have a broad substrate selectivity, notably the enzymes factor inhibiting HIF (FIH)^14–16^ and JMJD6.^6,17–19^ At least for FIH, evidence has been provided that it can catalyse oxidation reactions other than simple hydroxylation, *e.g.*, desaturation, and that it can oxidise residues other than its initially reported asparaginyl HIF-α substrate, including (2*R*)-asparaginyl, aspartyl, and, like MINA53/NO66, histidinyl C-3 hydroxylation substrates (Fig. 1d).^17,20–22^ FIH has also been shown to regulate the activity of OTUB1 with which it reacts to form a stable adduct.^23^ To date, however, there has been only limited understanding of the substrate selectivities of the ribosomal oxygenases MINA53/NO66 with respect to their C-3 histidine hydroxylase activity, a knowledge gap that we address in this study.

Here we report studies on the substrate selectivity and inhibition of MINA53 and NO66 employing peptide fragments of their natural Rpl substrates, wherein the substrate histidine residue was substituted by a range of natural and unnatural amino acid residues. By contrast with FIH, the results imply that MINA53 and NO66 have a narrow substrate selectivity, however, inhibition studies with the substrate analogue peptides reveal that selective modulation of their activity should be possible.

## Results and discussion

**Fig. 2:**
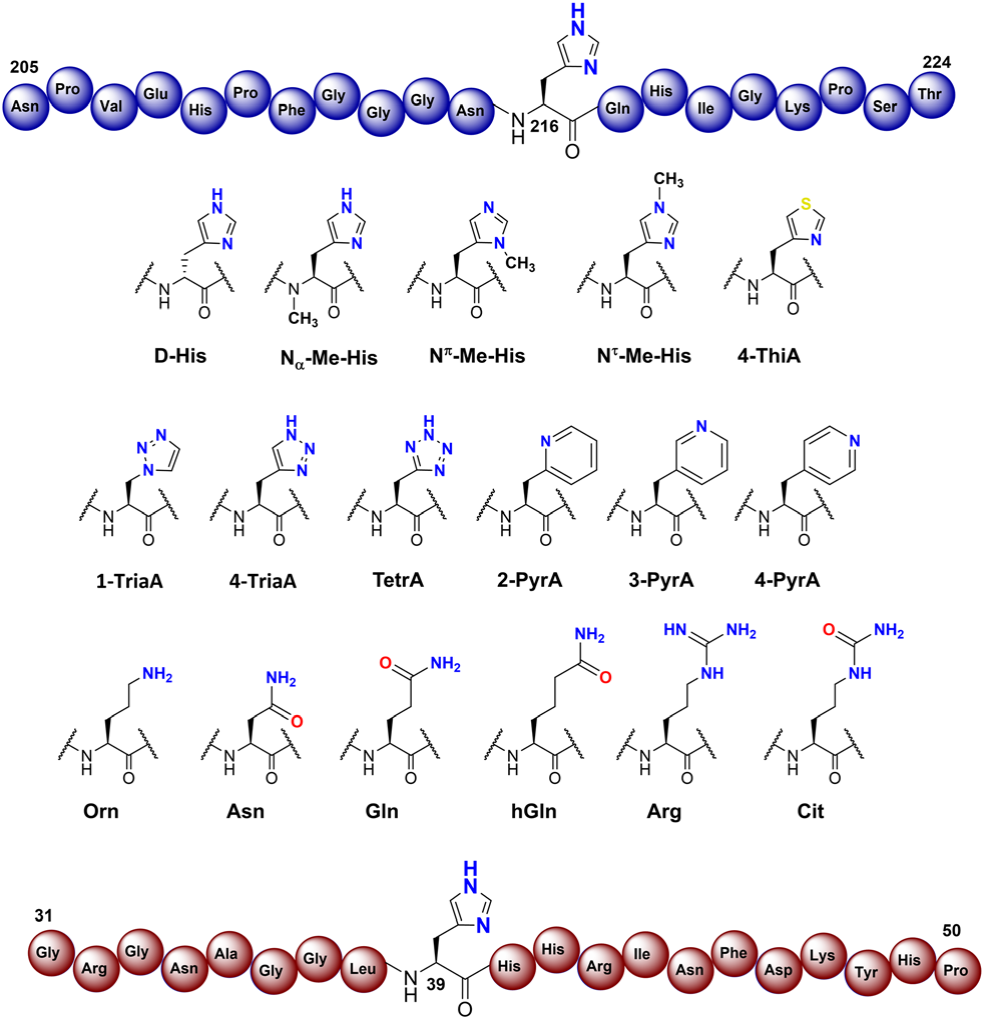
Histidine substrate analogues incorporated into Rpl8-His216 and Rpl27a-His39 sequences, respectively.

To investigate the substrate selectivity of human NO66/MINA53, we incorporated a panel of histidine analogues and other residues into 20-mer peptides of Rpl8 (residues 205-224, at position 216) and Rpl27a (residues 31-50, at position 39), respectively (Fig. 2). In addition to the natural sequences, analogues synthesised included: (a) the D-stereoisomer of histidine, (b) peptides with a methyl group at either N^π^ or N^τ^ of the histidine imidazole ring, or on the backbone amide nitrogen, (c) regioisomeric pyridine containing analogues, (d) addition of nitrogens to the imidazole ring, (e) exchange of nitrogen for sulphur at the imidazole N^τ^ position, and (f) side chain variations to introduce more conformationally flexible analogues. The peptides were prepared using standard microwave-assisted Fmoc-SPPS protocols and purified by reverse phase HPLC, followed by characterisation using matrix assisted laser desorption mass spectrometry (MALDI-TOF MS) and analytical HPLC (Tables S1 and S2, ESI†).

**Fig. 3:**
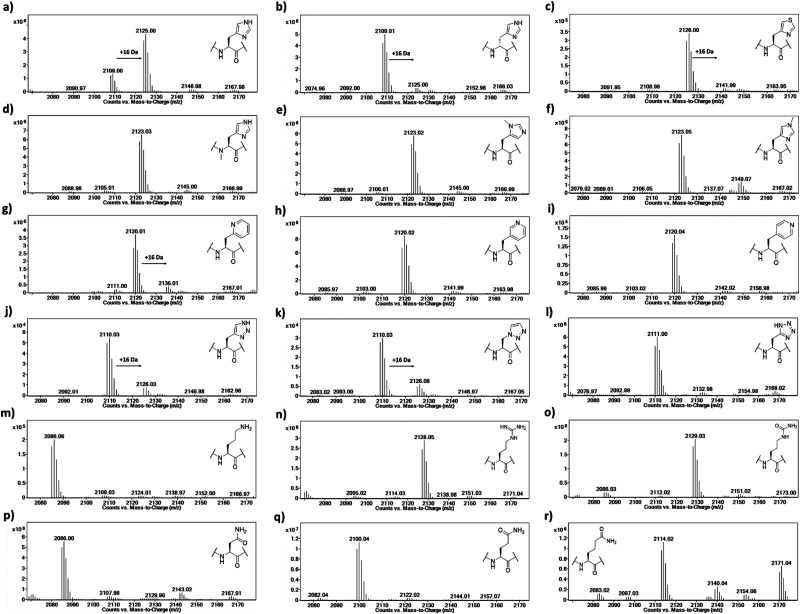
LC-MS data showing potential oxidation of Rpl8-His216 peptides in the presence of NO66. (a) Rpl8-His216, (b) Rpl8-D-His216, (c) Rpl8-ThiA216, (d) Rpl8-N_α_-His216, e) Rpl8-N^π^-me-His216, (f) Rpl8-N^τ^-Me-His216, (g) Rpl8-2PyrA216, (h) Rpl8-3PyrA216, (i) Rpl8-4PyrA216, (j) Rpl8-4-TriaA216, (k) Rpl8-1-TriaA216, (l) Rpl8-TetrA216, (m) Rpl8-Orn216, (n) Rpl8-Arg216, (o) Rpl8-Cit216, (p) Rpl8-Asn216, (q) Rpl8-Gln216 and (r) Rpl8-hGln216. Conditions: 2 μM NO66, 10 μM Rpl peptide, 100 μM L-ascorbate, 10 μM ferrous ammonium sulfate, 10 μM 2OG, pH 7.0, 2 hours at room temperature.

The Rpl peptides possessing histidine analogues were initially evaluated as MINA53 and NO66 substrates (2 μM MINA53/NO66, 10 μM Rpl peptide, 100 μM L-ascorbate, 10 μM ferrous ammonium sulfate, 10 μM 2OG) with incubation for 2 hours at pH 7.0 (NO66) or 7.5 (MINA53) at room temperature,^24^ with potential conversion to hydroxylated products being monitored by liquid chromatography-mass spectrometry (LC-MS) (Fig. 3 and Fig. S1, ESI†). Positive controls showed that, as anticipated, the Rpl27a-His39 and Rpl8-His216 peptides are efficiently hydroxylated (∼75% conversion) by MINA53 and NO66, respectively, under the assay conditions. None of the substrate analogues were efficient MINA53/NO66 substrates in our assays.

A low level of potential hydroxylation was detected for the Rpl27a-D-His39 (∼10%, Fig. S1b, ESI†) and Rpl8-D-His216 (∼5%, Fig. 3b) peptides, with MINA53 and NO66, respectively, an observation precedented by work on another JmjC hydroxylase FIH.^21^ Interestingly, the Rpl8-2PyrA216 peptide (Fig. 3g) manifested evidence for low level hydroxylation (∼20%) with NO66, but there was no evidence that the analogous Rpl27a-2PyrA39 peptide was a substrate for MINA53, implying differences in the active site of the two ROX enzymes (Fig. S1g, ESI†). Very low levels of potential hydroxylation were also observed for Rpl8-4-TriaA216 (Fig. 3j) and Rpl8-1-TriaA216 with NO66 (∼10%, Fig. 3k) at pH 7.0. For the rest of the panel of Rpl peptides, no conversion was observed. To investigate the role of protonation in binding processes required for productive catalysis, MALDI-TOF MS assays were conducted at pH 4.5, 6.0, and 7.5 for Rpl27a-His39 with MINA53 and Rpl8-His216 with NO66, along with the tetrazole and 4-triazole substrate analogues (Fig. S2 and S3, ESI†). In all cases, lowering the pH did not increase the extent of hydroxylation. Due to low hydroxylation levels of some NO66 and MINA53 substrates under standard conditions, steady state kinetic analyses were not carried out. Overall, these results imply MINA53 and NO66 have a narrower substrate residue selectivity than FIH.^21^

To further investigate hydroxylation efficiency, assays with MINA53 (2 μM) were carried out using Rpl27a peptides at 50 μM (Fig. S4 and S5, ESI†). Under these conditions a high level of hydroxylation was detected for Rpl27a-His39 and comparatively less for Rpl27a-D-His39. Rpl27a-4-ThiA39 was poorly hydroxylated (∼10%); notably, the Rpl27a-4-TriA39 triazole analogue underwent ∼55% hydroxylation under these conditions.

To confirm the site of hydroxylation in an unnatural histidine analogue containing Rpl peptide, MALDI-TOF based MS/MS analysis was carried out with the apparent NO66-catalysed hydroxylation product of Rpl8-4-TriaA216; this analogue was chosen for study because of its relatively high level of hydroxylation. To achieve sufficient hydroxylation, the Rpl8-4-TriaA216 peptide was incubated for 4 hours with a ratio of 1 : 2 with NO66, which resulted in ∼40% hydroxylation (Fig. S6, ESI†). Subsequently, both the unmodified and hydroxylated peaks were fragmented using MALDI-TOF MS/MS. In the substrate spectrum, the observation of a y_9_′ peak with *m*/*z* 1004.53 and y_10_′ peak with *m*/*z* 1118.58 is indicative of a lack of hydroxylation, as expected. In the product spectrum, new peaks for y_9_′ with *m*/*z* 1020.52 and y_10_′ with *m*/*z* 1134.57, supporting production of the hydroxylated Rpl8-4-TriaA216 peptide (Fig. S7 and S8, ESI†). +16 Da mass shifts were not observed for peaks correlating to fragments that did not possess the 4-TriaA216 residue, exemplified by the y_8_′ peak with *m*/*z* 866.48 (substrate) and 866.48 (product) being detected in the both MS/MS spectra with no evidence for a peak at *m*/*z* ∼882, indicating a lack of hydroxylation in the y_8_′ fragment (Fig. S7 and S8, ESI†) in both the substrate and product. Although further validation with more efficient substrate analogues is desirable, these observations support the proposal that, at least in the case of NO66-catalysed oxidation of Rpl8-4TriaA216, hydroxylation occurs at residue 216.

**Fig. 4:**
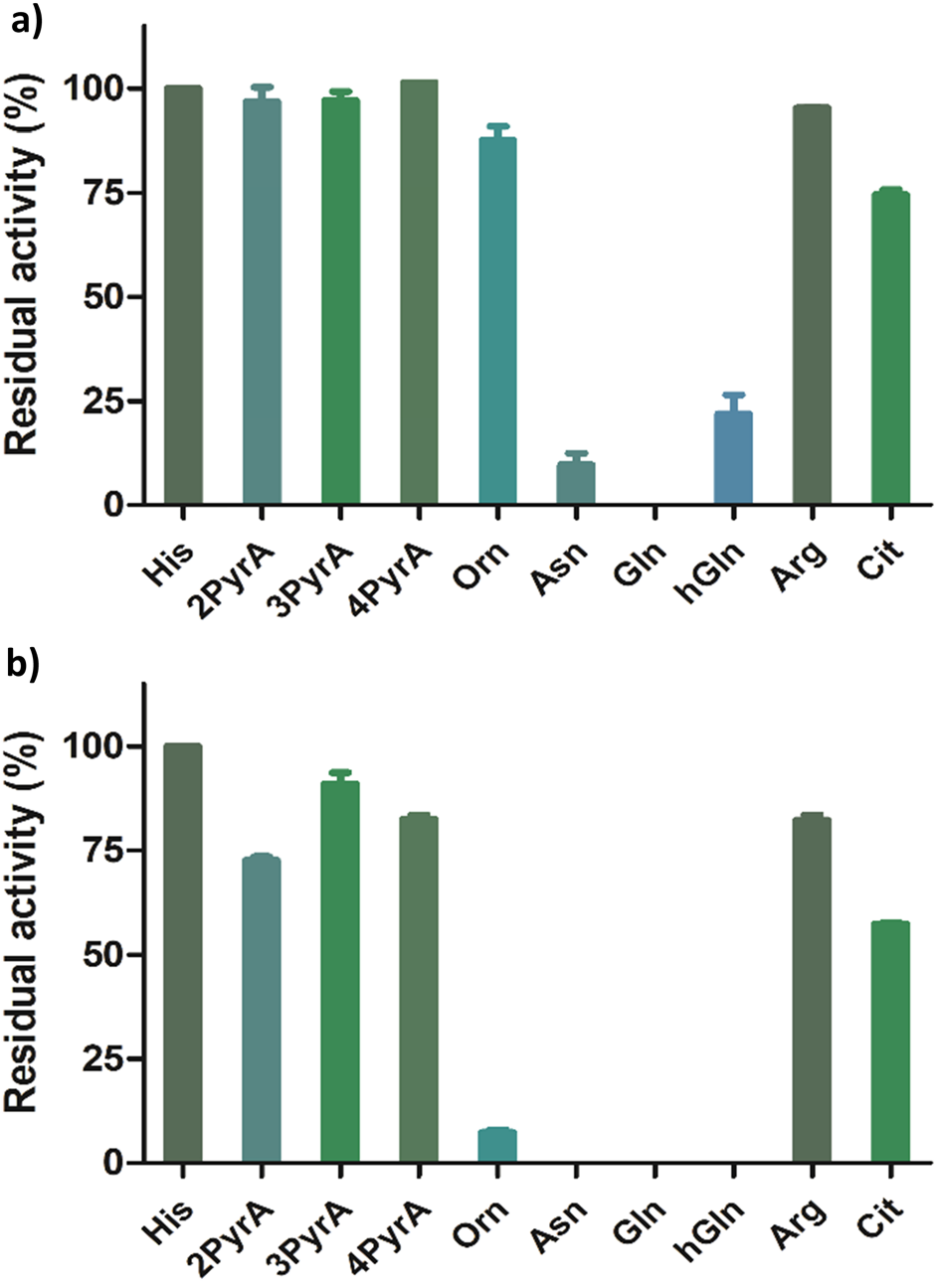
Results for MINA53 inhibition at (a) 5 μM and (b) 50 μM by Rpl27a peptides. Error bars reported as standard errors (SE) carried out in duplicate. See Fig. 2 for analogue structures.

Having shown that our Rpl peptides are not, at least efficient, ROX substrates, we explored them as inhibitors of the recombinant human ROXs using MALDI-TOF assays (Rpl8/27a-N^τ^-Me, Rpl8/27a-N^π^-Me, Rpl8/27a-1-TriaA, Rpl8/27a-4-TriaA and Rpl8/27a-TetrA were excluded from the panels for NO66 and MINA53, respectively, due to signal overlap between the two peptides in the assays). Synthetic Rpl peptides (5 or 50 μM) were incubated with MINA53/NO66 (400 nM), ferrous ammonium sulfate (10 μM) and 2OG (10 μM) for 10 minutes, after which time the Rpl27a-His39 and Rpl8-His216 substrates were added, respectively, and the reactions were carried out for 2 hours at room temperature (Fig. 4 and Fig. S9, ESI†). For MINA53, the unnatural Rpl27a-His39 inhibitors bearing 2PyrA, 3PyrA, 4PyrA residues did not manifest evidence for inhibition, even at 50 μM (Fig. 4). The Asn, Gln and hGln peptides, however, displayed inhibition of MINA53 at 5 μM. Use of a 10-fold increase of the inhibitors (50 μM) showed strong inhibition with the Orn, Asn, Gln, hGln possessing Rpl27a peptides (Fig. 4). By contrast, for NO66, the 2PyrA, 3PyrA, 4PyrA, Orn, Arg and Cit containing Rpl8 peptides did not show any inhibition activity, even at 50 μM (Fig. S9, ESI†). It is also worth noting that none of the Rpl peptides (at 50 μM) were observed to be hydroxylated in the inhibition assays in the presence of NO66 and MINA53, indicating that they act as inhibitors and not as substrates. Amongst the amide containing amino acid Rpl8 peptides, Asn and hGln were shown to display significant NO66 inhibition activity (∼50% inhibition at 5 μM). Given that the bacterial homologue (Ycfd) of MINA53/NO66, catalyses C3-hydroxylation of an arginine-residue in the ribosomal protein L16,^5,8^ it is of interest that the Rpl27a and Rpl8 analogues with arginine substituted for histidine did not inhibit MINA53 or NO66, respectively.

**Fig. 5:**
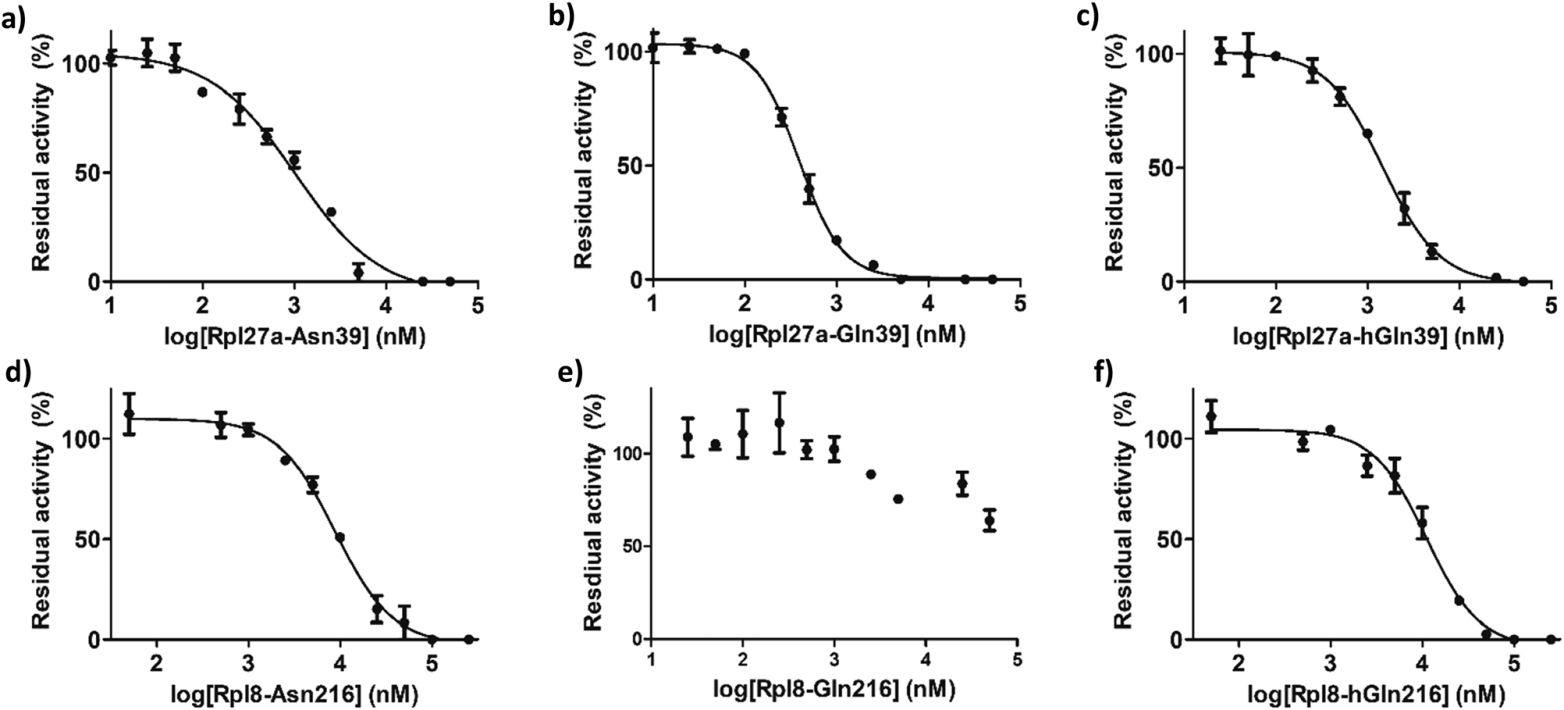
Dose-response curves showing inhibition of MINA53-catalysed Rpl27a-His39 by (a) Rpl27a-Asn39, (b) Rpl27a-Gln39 and (c) Rpl27a-hGln39 peptides; and NO66-catalysed Rpl8-His216 by (d) Rpl8-Asn216, (e) Rpl8-Gln216 and (f) Rpl8-hGln216 peptides. Error bars are reported as SE for duplicate assays.

IC_50_ values were determined for the peptides that manifested clear inhibition potency by varying the concentration of the Rpl peptide inhibitor from 10 nM to 250 μM (Fig. 5 and Table 1). Rpl27a-Gln39 was found to be the most potent MINA53 inhibitor (IC_50_ 0.39 μM), but the Rpl27a-Asn39 (IC_50_ 1.02 μM) and the Rpl27a-hGln39 (IC_50_ 1.47 μM) peptides were also potent MINA53 inhibitors. Strikingly, with NO66 the analogous three Rpl peptides were much weaker inhibitors, *i.e.*, Rpl8-Asn216 had an IC_50_ of 8.86 μM, Rpl8-hGln216 an IC_50_ 11.2 μM, and that of Rpl8-Gln216 > 100 μM.

IC_50_ values for MINA53 inhibition by Rpl27a peptides and NO66 inhibition by Rpl8 peptides. Conditions: 400 nM MINA53/NO66, 10 nM to 250 μM inhibitor Rpl peptide, 100 μM L-ascorbic acid, 10 μM ferrous ammonium sulfate, 10 μM 2OG in 50 mM MES buffer (pH 7.0) for NO66 and 50 mM HEPES (pH 7.5) for MINA53

**Table:** 

MINA53	IC_50_ (μM)
Rpl27a-Asn39	1.02 ± 0.11
Rpl27a-Gln39	0.39 ± 0.16
Rpl27a-hGln39	1.47 ± 0.27

**Table:** 

NO66	IC_50_ (μM)
Rpl8-Asn216	8.86 ± 0.36
Rpl8-Gln216	>100
Rpl8-hGln216	11.2 ± 0.40

**Fig. 6:**
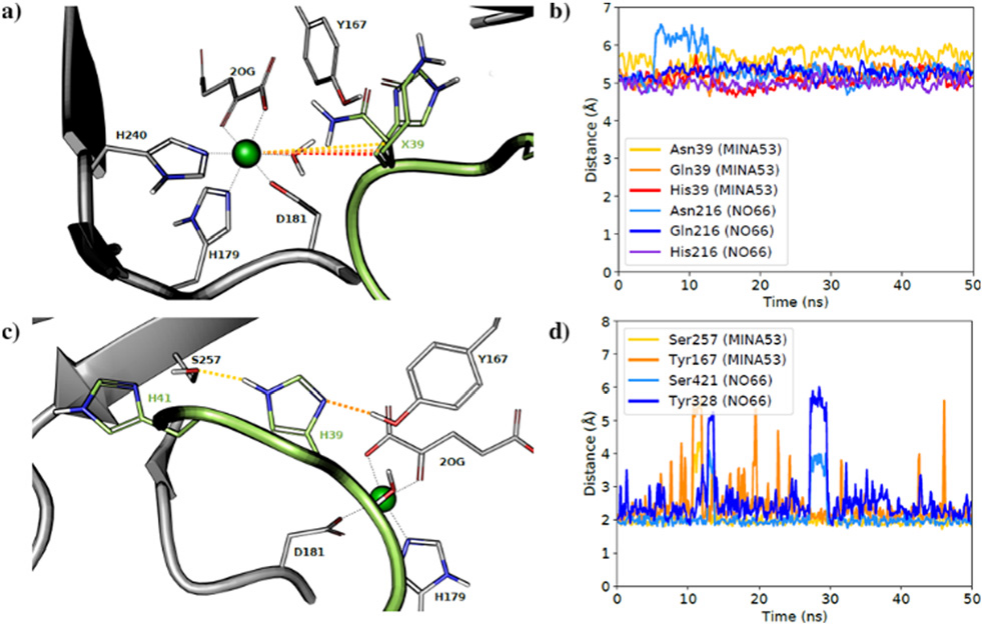
Molecular dynamics simulations. (a) Average aligned coordinates from 50 ns MD simulations of MINA53 with different substrates based on a crystal structure (PDB: 4BXF). Dotted lines indicate distances between the β-carbon and the active site ion; (b) distance between the β-carbon and the iron in MD simulations of NO66 and MINA53 with listed different Rpl peptides; (c) average coordinates from a MD simulation of MINA53 based on a crystal structure (PDB: 4BXF). Dotted lines indicate H-bonds to the substrate, His39 (Rpl27a); (d) H-bond distance between His39(Rpl27a), His216(Rpl8) and neighbouring Ser257/Ser421 and Tyr167/Tyr328 residues in the MD simulations of MINA53 and NO66 in complex with the Rpl analogues.

To investigate substrate analogue binding, a series of 50 ns molecular dynamics (MD) simulations of MINA53 and NO66 bound to different selected Rpl8(His216)/Rpl27(His39) analogues (Asn39/Asn216, Gln39/Gln216) were performed. To compare substrate analogue positioning during the MD simulations, the distances between the ferrous iron cofactor and the potentially hydroxylated β-carbon of the substrate residue were measured (Fig. 6a and b). These analyses showed that the positioning of the β-carbon remains relatively undisturbed, at around 5–6 Å, including for the natural histidine substrates, during most of the simulations for all the Rpl peptides, suggesting that binding of all the investigated Rpl peptides is stable, supporting the experimental findings.

The natural substrate residues, that is His39(Rpl27a)/MINA53 and His216(Rpl8)/NO66, take part in a proton-sharing network with neighbouring Tyr167/Tyr328 and Ser257/Ser421 enzyme residues for MINA53 and NO66, respectively (Fig. 6). The serine residues Ser257/MINA53 and Ser421/NO66 are positioned to form a hydrogen bond with the His41(Rpl27a)/His218(Rpl8) histidine residues to complete the hydrogen bond network. Analysis of the hydrogen bond distances between His39(Rpl27a)/His216(Rpl8) and the Ser257/Ser421 and Tyr167/Tyr328 MINA53/NO66 residues during the MD simulations implies that both hydrogen bonds are stable during most of the simulations (Fig. 6c and d).

It is likely that the aforementioned hydrogen bonds are critical for the optimal binding and orientation of His39(Rpl27a)/His216(Rpl8) substrate residues for productive catalysis, which likely involves the substrate C-3 carbon moving closer to the ferrous ion cofactor than observed in the MD simulations. For both the MINA53 and NO66 complexes, we observed that the hydrogen bond distance to Ser257/Ser421 for MINA53/NO66 was the most stable. Glutamine and asparagine residues are in principle capable of taking part in the same hydrogen bond network as does histidine, which undergoes hydroxylation. Analysis of the average structures from the MD simulations (Fig. S10, ESI†) for Gln/Asn/His residues at the potentially hydroxylated positions reveals clear differences in their predicted binding modes. Gln39(Rpl27a)/Gln216(Rpl8) were observed to form hydrogen bonds with both the Tyr167/Tyr328 and Ser257/Ser421 MINA53/NO66 residues, in a similar manner to His39(Rpl27a) and His216(Rpl8). By contrast, the side chains of Asn39(Rpl27a)/Asn216(Rpl8) were observed to be too short to allow hydrogen bonding to Ser257/Ser421. As a result, Asn39/Asn216 were observed to only form hydrogen bonds with Tyr167/Tyr328 of MINA53/NO66.

The overall MD simulation results suggest that the Gln, Asn and hGln inhibiting analogues bind in a similar manner to the His substrate residue; however, they do not explain the differences in selectivity of inhibition, notably that Rpl27a-Gln39 is a potent inhibitor of MINA53, but Rpl8-Gln216 does not inhibit NO66, an observation that requires further investigation.

## Conclusions

At least one of the human 2OG dependent protein hydroxylases, *i.e.*, FIH, appears promiscuous in terms of the proteins and residues it can accept as substrates,^21^ whereas another JmjC hydroxylase, JMJD6, can accept multiple proteins, but its assigned activity is presently limited to lysine hydroxylation and more controversially, N-methyl arginine residues demethylation.^6^ Some of the JmjC KDMs can also accept different N^ε^-methyllysine methylation states and act at different histone residues; some of the JmjC KDMs have also been shown to catalyse *N*-methylarginine residue demethylation, at least in isolated form.^25^ There are mixed reported results in terms of selectivity for protein hydroxylases from other 2OG-dependent oxygenase structural subfamilies. Thus, AspH, a C-3 hydroxylase acts on both aspartyl and asparaginyl residues in multiple epidermal growth factor like domains, whereas the hypoxia inducible factor prolyl-hydroxylases appear highly selective, at least in isolated form.^26,27^

The overall results with synthetic analogues of the Rpl27a and Rpl8 substrates of the human ribosomal oxygenases MINA53 and NO66, which catalyse the stereospecific C-3 hydroxylation of histidine residues in ribosomal proteins, indicate that they are highly selective for histidine residues, at least in terms of naturally occurring residues. Using relatively high enzyme:substrate ratios, we accrued evidence that MINA53 and NO66 can, albeit inefficiently (at least with the tested substrates) catalyse hydroxylation of D-His residues, with NO66 also catalysing inefficient hydroxylation of triazoles and sterically more demanding 2PyrA analogue. Although it is possible that MINA53/NO66 accept non-histidine residues within the context of proteins or other sequences, the available evidence is that they are highly selective with respect to the substrates they act on. Thus, it is of interest that substrate-based peptides can be potent inhibitors of MINA53 over NO66, as strikingly observed for the Rpl27a and Rpl8 analogues with His substituted for Gln, where potent inhibition of MINA53 (by Rpl27a-Gln39), but not of NO66 (by Rpl8-Gln216), was observed. Further, the Asn and hGln analogues were more potent inhibitors of MINA53 than NO66. The potency of MINA53 inhibition by the Rpl27a peptides was similar to that of recently reported small-molecule MINA53 inhibitors.^24^ The precise mode of inhibition of the peptide inhibitors of MINA53 and NO66 is the subject of ongoing biophysical analyses – the results should help in the identification of more potent and selective MINA53/NO66 inhibitors. It is notable that the most potent inhibition of MINA53/NO66 was observed with peptides with acyclic side chains, implying the conformational constraints for inhibition are less strict than for catalytic oxidation. It is also of interest that Arg containing substrate analogues were neither inhibitors nor substrates of MINA53/NO66, because a bacterial homologue of them (YcfD) catalyses C-3 hydroxylation of an arginine-residue in the ribosomal protein L16.^5,8^ The inhibition of MINA53 and NO66 by the Asn containing analogues is interesting given that multiple proteins with Asn residues are substrates for other 2OG oxygenases, *i.e.*, FIH and AspH.^26,27^

The overall inhibition results, including with Rpl peptides containing histidine analogues with acyclic occurring side chain (Asn, Gln), raise the possibility that the activities of MINA53/NO66 and, by implication other 2OG dependent protein hydroxylases/demethylases, might be regulated *in vivo* by competition with non-oxidised protein/peptide substrate analogues.

Finally, we note the results with histidine substrate analogues in this work highlight the value of using of unnatural amino acid residues in examinations of biomolecular recognition and biocatalysis, as also used for probing lysine posttranslational modifications^28^ and in studies on 2OG oxygenases acting on small molecules, *e.g.*, in antibiotic biosynthesis.^29^ Together with recent work on the histidine methyltransferase SETD3,^30^ the current results demonstrate that histidine-modifying enzymes display clearly different substrate specificities.

## Experimental

### Peptide synthesis and purification

The Rpl27a_31-49_ (GRGNAGGLHHHRINFDKYHP) and Rpl8_205-224_ (NPVEHPFGGGNHQHIGKPST) 20-mer peptides were assembled using Rink amide resin until position His216 of Rpl8_205-224_ and His39 of Rpl27a_31-49_. The remainder of the sequences were assembled using microwave assisted SPPS on a Liberty Blue peptide synthesiser (CEM corporation, Matthews, NC, USA). Amino acid couplings were carried out with the molar ratio of (5) : (5) : (7.5) of (Fmoc-protected amino acid) : (DIC) : (Oxyma Pure) at 75 °C for 2 min. (Un)natural amino acids were coupled by manual SPPS with the equivalent ratio (5) : (5) : (7.5) of (Fmoc protected amino acid) : (COMU) : (DIPEA) overnight at room temperature. Other couplings were by manual SPPS the molar equivalent ratio (5) : (5) : (7.5) of (Fmoc protected amino acid) : (HATU) : (DIPEA) for 1 hour at room temperature and deprotection in 20% (v/v) piperidine for 30 min at room temperature. The peptide proceeded to standard cleavage from resin using 2.5% (v/v) TIPS, 2.5% (v/v) H_2_O in conc. CF_3_COOH for 3 hours. CF_3_COOH was removed using N_2_ and the resultant residue suspended in cooled Et_2_O. After suspension, the mixture was centrifugated (5 min, 5000 rpm) in an Eppendorf 5804R centrifuge (Eppendorf, Hamburg, Germany) after which the supernatant was decanted into the waste. The remaining solid was washed twice by cold Et_2_O and subjected to centrifugation. The crude peptide was dissolved in a mixture of MeCN in H_2_O, then purified using RP-HPLC and a gradient of H_2_O + 0.1% (v/v) CF_3_COOH and MeCN + 0.1% (v/v) CF_3_COOH from 10% (v/v) MeCN to 100% (v/v) MeCN over 40 min at 4 mL min^−1^ using Gemini 10 μm NX-C18 110. LC column (Phenomenex, Torrance, CA, USA). For some peptides, including Rpl8-4-ThiA39, Orn, 4-TriaA39, 1-TriaA39, Cit and Rpl27a-1-TriaA39, from 20% (v/v) MeCN + 0.1% CF_3_COOH to 60% (v/v) MeCN + 0.1% (v/v) CF_3_COOH over 15 min, followed by 100% (v/v) MeCN + 0.1% (v/v) CF_3_COOH in 10 min over 40 min at 4 mL min^−1^ was used. Rpl8-N^τ^-me-His216 was purified using 3% (v/v) MeCN + 0.1% (v/v) CF_3_COOH to 40% (v/v) MeCN + 0.1% (v/v) CF_3_COOH over 25 min, then 100% (v/v) MeCN + 0.1% (v/v) CF_3_COOH from 26–29 min over 30 min at 10 mL min^−1^. Analytical RP-HPLC employed a Gemini 5 μm C18 110. LC column (Phenomenex) at a flow rate of 1 mL min^−1^ with a gradient of H_2_O + 0.1% (v/v) CF_3_COOH and MeCN + 0.1% (v/v) CF_3_COOH from 3% (v/v) MeCN to 100% MeCN + 0.1% (v/v) CF_3_COOH over 30 min at 1 mL min^−1^. Analytical spectra were monitored at 215 nm.

### In-solution click chemistry

Rpl8-1-TriaA216, Rpl27a-1-TriaA39, Rpl8-4-TriaA216 and Rpl27a-4-TriaA39 were synthesised by the use of Fmoc-Dap(N3)-OH and Fmoc-N-(propargyl)-glycine-OH on His39 and His216 for Rpl8 and Rpl27a, respectively, as reported.^30^ The click reagent (NaN_3_ or TMS-acetylene, 45 equiv.) was dissolved in 300 μL Milli-Q water and then added to the Rpl27a_31-49_/Rpl8_205-224_ peptide (1 equiv.) followed by brief mixing by vortexing. CuSO_4_ (6 equiv.) was then dissolved in 200 μL Milli-Q water and mixed with *tert*-butylimino-tri (pyrrolidino)phosphorane (BTTP) (2.6 equiv.) followed by addition of sodium L-ascorbate (4 equiv.) in 200 μL Milli-Q water. This mixture was added to the peptide solution, followed by the addition of 40 μL in *N*,*N*-diisopropylethylamine (DIPEA), to give a mixture that was reacted overnight at room temperature with shaking. The mixture was then diluted with 300 μL MeCN and directly purified by RP-HPLC using a gradient of buffer A and buffer B from 10% B to 100% (v/v) over 40 minutes at 4 mL min^−1^, fractions containing the purified product were collected then lyophilized.

### MALDI-TOF measurements

The Rpl peptide was measured by MALDI-TOF MS using a mixture of α-cyano-4-hydroxycinnamic acid (CHCCA) matrix mixed in a mixture of purified water and MeCN (1 : 1, v/v in MeCN/MilliQ) and loaded onto an MTP 384 polished steel target to be analysed by a MALDI-TOF UltrafleXtreme-II tandem mass spectrometer (Bruker). MALDI-TOF MS/MS fragmentation was achieved using a timsTOF flex MALDI-2 machine (Bruker). MS/MS spectra were analysed using mMass (https://www.mmass.org) and peptide fragmentation lists were generated using GPMAW 13 (https://www.gpmaw.com/html/downloads.html).

### Recombinant protein production and purification

The MINA53 coding sequence (A26-V464) and NO66 coding sequence (A167-N641) were sub-cloned into an expression vector pET-28b respectively and the plasmids were transformed into Escherichia coli strain BL21(DE3).^5,24^ In brief, a 6 × 10 mL overnight culture was used to inoculate 6 l of Terrific Broth media containing 100 μg mL^−1^ kanamycin. Cultures were grown at 37 °C until the OD_600_ reached ∼1.0. The temperature was adjusted to 18 °C, and expression was then induced or 18 hr with 0.5 mM isopropylthio-β-galactoside (IPTG). Cells were centrifuged, then resuspended in the lysis buffer (50 mM HEPES pH 7.4, 500 mM NaCl, 20 mM imidazole, 0.5 mM tris-(2-carboxyethyl)-phosphine (TCEP), and 5% glycerol in the presence of a protease inhibitor mixture 1 : 2000 (Complete, EDTA-free Protease Inhibitor Cocktail, Roche Diagnostics Ltd) and lysed by three passages through a high-pressure cell breaker (EmulsiFlex C5-Avestin) at 4 °C. The lysates were cleared by centrifugation (60 minutes, 36 000× g, 4 °C) and loaded onto a Ni NTA column. After extensively rinsing with the lysis buffer the His6-tagged MINA53 and NO66 proteins were eluted using lysis buffer containing 300 mM imidazole. The eluted fractions were further purified using an AKTA Xpress system combined with an S200 gel filtration column equilibrated in 20 mM HEPES (pH 7.4), 150 mM NaCl, 0.5 mM tris (2-carboxyethyl)-phosphine (TCEP) and 5% glycerol. The purity was confirmed by SDS-PAGE and by mass spectrometry as reported.^5,24^

### Enzymatic assays

All reagents were from Sigma Aldrich and of the highest grade available. Ferrous ammonium sulphate (FAS) solution was prepared freshly by dissolving FAS in 400 mM in 20 mM HCl with subsequent diluted to 1 mM using deionized water. 2-Oxoglutarate (2OG, 10 mM) and L-ascorbic Acid (LAA, 50 mM) solutions were prepared freshly by dissolving the solids in deionised water. MINA53 and NO66 assays using Rpl27a_31-49_ and Rpl8_205-224_ peptides, respectively, were performed with the following conditions: 2 μM MINA53/NO66, 10 μM Rpl peptide, 100 μM LAA, 10 μM FAS, 10 μM 2OG and 2 hours incubation at room temperature in 50 mM MES buffer (pH 7.0) for NO66 and 50 mM HEPES buffer (pH 7.5) for MINA53.^24^ All enzyme reactions were initiated by transfer of the appropriate enzyme to 200 μL of the cosubstrate/substrate solution with incubation at room temperature for 2 hours. A positive control reaction containing enzyme and a validated peptide substrate was included in all screening studies. After 2 hours the enzyme reaction was stopped by addition of 20 μL of 10% (v/v) LC-MS grade formic acid (Fisher Scientific) and the reactions transferred to a 96 well plate (Agilent). Intact mass peptide analysis was performed by Liquid Chromatography-Mass Spectrometry (LC-MS) using an Agilent 1290 infinity II LC system equipped with an Agilent 1290 multisampler and an Agilent 1290 high speed pump and connected to an Agilent 6550 accurate mass iFunnel quadrupole time of flight (Q-TOF) mass spectrometer. 10 μL of enzyme reaction were injected and loaded onto a ZORBAX RRHD Eclipse Plus C18 column (Agilent). Solvent A consisted of LC-MS grade water containing 0.1% (v/v) formic acid and solvent B consisted of MeCN containing 0.1% (v/v) formic acid. Peptides were separated using a step wise gradient (0 min – 95% solvent A, 1.0 min – 80% solvent A, 3.0 min – 45% solvent A, 4.0 min – 45% solvent A, 5.0 min – 0% solvent A, 6.0 min – 0% solvent A, 7.0 min – 95% solvent A). This was followed by a 3 min elution with 95% solvent A to re-equilibrate the column. Flow rates were 0.2 mL min^−1^. The mass spectrometer was operated in the positive ion mode with a drying gas temperature (280 °C), drying gas flow rate (13 L min^−1^), nebulizer pressure (40 psig), sheath gas temperature (350 °C), sheath gas flow rate (12 L min^−1^), capillary voltage (4000 V), nozzle voltage (1000 V). All acquired data were analysed using Agilent MassHunter Qualitative Analysis (Version B.07.00) software.

### Inhibition assays

MINA53 and NO66 inhibition assays in the presence of Rpl27a_31-49_ and Rpl8_205-224_ peptides, respectively, were performed under the following assay conditions: 400 nM MINA53/NO66, 5/50 μM inhibitor Rpl peptide, 100 μM LAA, 10 μM FAS, 10 μM 2OG in 50 mM MES buffer (pH 7.0) for NO66 and 50 mM HEPES buffer (pH 7.5) for MINA53.^24^ After 10 minutes of preincubation 5 μM of substrate peptide was added and the reaction was incubated for 2 hours at room temperature. A positive control reaction containing enzyme and unmodified peptide substrate without any inhibitors was included. After 2 hours the reaction was stopped by addition of 20 μL of 10% (v/v) LC-MS grade formic acid (Fisher Scientific), mixed 1 : 1 with α-cyano-4-hydroxycinnamic acid (CHCCA) matrix dissolved in a mixture of H_2_O and MeCN (1 : 1, v/v), and loaded onto an MTP 384 polished steel target to be analyzed by MALDI-TOF MS. MINA53/NO66 residual activity was determined by calculating the relative integral of the methylated peptide to a control reaction in absence of potential inhibitory peptides. Experiments were carried out in duplicate.

### System preparation and MD simulations

The structures of NO66 (PDB ID: 4Y3O)^31^ and MINA53 (PDB ID: 4BXF)^6^ were imported into the Protein Preparation Wizard in Maestro.^32^ The Protein Preparation Wizard was used to add hydrogens, determine bond orders, determine protonation states, and fill in missing loops. Protonation states were determined at pH = 7 using Epik; whereas the loops were introduced using Prime.^33,34^ Chain A and C was then removed from the NO66 structure, while chains B and D were extracted from the MINA53 structure. Both entries were then exported. General Amber Force Field (GAFF2) parameter files for 2-oxoglutaric acid (2OG) and N-oxalylglycine (NOG) were built using the AM1-BCC charge method in Antechamber, which is available in Amber.^35–37^ Bonded parameters for iron atoms were developed using the Metal Center Parameter Builder (MCPB.py).^38,39^ Bonds between iron and water were initially removed and reintroduced as harmonic distance restraints in Amber.

Tleap was used to substitute Cys6 in the MINA53 sequence to glycine and introduce different analogues of the substrate histidine residues in the substrate sequence. Tleap was used to add missing side chain atoms and solvate the systems in TIP3P water boxes with a NaCl concentration of 0.150 M and a surplus of Na^+^ ions to neutralize the systems.^40^ The FF14SB force field was used to provide protein parameters.^41^

MD simulations were performed using Amber and Particle Mesh Ewald (PME), with a 10.0 Å nonbonded cutoff, a 2 fs time step, and the SHAKE algorithm to constrain bonds involving hydrogen.^42–44^ Six MD simulations were performed in total: three of MINA53 and three of NO66 in complex with different substrate/substrate analogue peptides (with His39, Asn39, and Gln39, at the position of the hydroxylated residue). The systems were initially minimized for 500 steps using the steepest descent algorithm followed by 500 steps using the conjugate gradient algorithm. The minimizations were performed with restraints on the protein backbone. The systems were then annealed from 0 K to 300 K for 50 ps in the *NVT* ensemble using the Langevin thermostat while maintaining the backbone restrains.^45^ After this, the Berendsen barostat was applied to control the pressure. After 50 ps of simulation in the NPT ensemble, the restraints were lifted, and a final 50.5 ns production simulation was performed. The first 500 ps of the production simulations were considered as system equilibration and removed before analysis.

## Author contributions

J. M. and C. J. S. conceived and supervised the project. V. A. T. synthesised and purified Rpl peptides. J. C. J. H. carried out inhibition assays. A. T. carried out enzyme assays. L. M. and J. K. performed M. D. simulations. E. S. produced proteins. V. A. T., J. C. J. H., C. J. S. and J. M. wrote the manuscript.

## Conflicts of interest

There are no conflicts of interest.

## Supplementary Material

CB-004-D2CB00182A-s001
