# Supplementary material for: Substrate selectivity and inhibition of histidine JmjC hydroxylases MINA53 and NO66
Source: RSC Chem Biol. 2023 Jan 12;4(3):235–43. doi: 10.1039/d2cb00182a (PMC9994133; doi:10.1039/d2cb00182a)
Supplement: CB-004-D2CB00182A-s001 [file CB-004-D2CB00182A-s001.pdf]

Electronic Supplementary Material (ESI) for RSC Chemical Biology.
This journal is © The Royal Society of Chemistry 2023

Supplementary Information

Substrate selectivity and inhibition of histidine JmjC hydroxylases MINA53 and NO66

Vildan A. Türkmen,[a] Jordi C. J. Hintzen,[a] Anthony Tumber,[b] Laust Moesgaard,[a] Eidarus Salah,[b] Jacob Kongsted,[a] Christopher J. Schofield,*[b] and Jasmin Mecinović*[a]

[a] Department of Physics, Chemistry and Pharmacy, University of Southern Denmark, Campusvej 55, 5230 Odense, Denmark

[b] Department of Chemistry and the Ineos Oxford Institute for Antimicrobial Research, Chemistry Research Laboratory, University of Oxford, 12 Mansfield Road, OX1 3TA Oxford, United Kingdom

Table of Contents

1. Characterisation of Rpl peptides 3

2. Turnover assays with MINA53 and NO66 5

3. Inhibition supporting figure 13

4. Molecular dynamics simulations 13

1. Characterisation of Rpl peptides

Table S1. Characterisation of Rpl27a peptides. All peptides were prepared with C-terminal amides.

Entry	Peptide	Sequence	Formula	m/z Calculated	m/z Found	Purity
1	(L)His39	GRGNAGGLHHHRINFDKYHP	$C_{100}H_{148}N_{38}O_{25}$	2282.15	2282.14	>99%
2	(D)His39	GRGNAGGLHHHRINFDKYHP	$C_{100}H_{148}N_{38}O_{25}$	2282.15	2282.19	>99%
3	N_{α}-Me-His39	GRGNAGGLHHHRINFDKYHP	$C_{101}H_{150}N_{38}O_{25}$	2296.16	2297.62	>99%
4	2PyrA39	GRGNAGGLHHHRINFDKYHP	$C_{102}H_{149}N_{37}O_{25}$	2293.15	2293.28	~94%
5	4ThiA39	GRGNAGGLHHHRINFDKYHP	$C_{100}H_{147}N_{37}O_{25}S$	2299.11	2299.81	~98%
6	N^{τ}-Me-His39	GRGNAGGLHHHRINFDKYHP	$C_{100}H_{148}N_{38}O_{25}$	2296.16	2296.88	>99%
7	N^{π}-Me-His39	GRGNAGGLHHHRINFDKYHP	$C_{100}H_{148}N_{38}O_{25}$	2296.16	2298.06	>99%
8	3PyrA39	GRGNAGGLHHHRINFDKYHP	$C_{102}H_{149}N_{37}O_{25}$	2293.15	2295.04	~98%
9	4PyrA39	GRGNAGGLHHHRINFDKYHP	$C_{102}H_{149}N_{37}O_{25}$	2293.15	2293.94	>99%
10	TetrA39	GRGNAGGLHHHRINFDKYHP	$C_{98}H_{146}N_{40}O_{25}$	2284.14	2293.94	>99%
11	4-TriaA39	GRGNAGGLHHHRINFDKYHP	$C_{99}H_{147}N_{39}O_{25}$	2283.14	2284.14	~98%
12	Orn39	GRGNAGGLHHHRINFDKYHP	$C_{99}H_{151}N_{37}O_{25}$	2259.17	2260.43	~95%
13	1-TriaA39	GRGNAGGLHHHRINFDKYHP	$C_{98}H_{146}N_{40}O_{25}$	2283.14	2283.15	>99%
14	Gln39	GRGNAGGLHHHRINFDKYHP	$C_{99}H_{149}N_{37}O_{26}$	2274.51	2273.17	>99%
15	hGln39	GRGNAGGLHHHRINFDKYHP	$C_{100}H_{151}N_{37}O_{26}$	2288.54	2287.23	>99%
16	Arg39	GRGNAGGLHHHRINFDKYHP	$C_{100}H_{153}N_{39}O_{25}$	2302.57	2302.41	>99%
17	Cit39	GRGNAGGLHHHRINFDKYHP	$C_{100}H_{152}N_{38}O_{26}$	2303.56	2302.40	>99%
18	Asn39	GRGNAGGLHHHRINFDKYHP	$C_{98}H_{149}N_{37}O_{26}$	2259.49	2259.18	>99%

Table S2. Characterisation of Rpl8 peptides. All peptides were prepared with C-terminal amides.

Entry	Peptide	Sequence	Formula	m/z Calculated	m/z Found	Purity
1	(L)His216	NPVEHPFGGGN<u>H</u>QHIGKPST	$C_{92}H_{136}N_{30}O_{28}$	2110.01	2109.52	>99%
2	(D)His216	NPVEHPFGGGN<u>H</u>QHIGKPST	$C_{92}H_{136}N_{30}O_{28}$	2110.01	2110.81	~95%
3	N_{α}-Me-His216	NPVEHPFGGGN<u>H</u>QHIGKPST	$C_{93}H_{138}N_{30}O_{28}$	2124.03	2123.65	~95%
4	2PyrA216	NPVEHPFGGGN<u>H</u>QHIGKPST	$C_{94}H_{137}N_{29}O_{28}$	2121.02	2120.50	>99%
5	4ThiA216	NPVEHPFGGGN<u>H</u>QHIGKPST	$C_{92}H_{135}N_{29}O_{28}S$	2126.98	2126.01	>99%
6	N^{τ}-Me-His216	NPVEHPFGGGN<u>H</u>QHIGKPST	$C_{93}H_{138}N_{30}O_{28}$	2124.03	2123.39	>99%
7	N^{π}-Me-His216	NPVEHPFGGGN<u>H</u>QHIGKPST	$C_{93}H_{138}N_{30}O_{28}$	2124.03	2123.02	>99%
8	3PyrA216	NPVEHPFGGGN<u>H</u>QHIGKPST	$C_{94}H_{137}N_{29}O_{28}$	2121.02	2120.72	>99%
9	4PyrA216	NPVEHPFGGGN<u>H</u>QHIGKPST	$C_{94}H_{137}N_{29}O_{28}$	2121.02	2120.72	~95%
10	TetrA216	NPVEHPFGGGN<u>H</u>QHIGKPST	$C_{90}H_{134}N_{32}O_{28}$	2112.00	2120.72	~97%
11	4-TriaA216	NPVEHPFGGGN<u>H</u>QHIGKPST	$C_{91}H_{135}N_{31}O_{28}$	2112.01	2111.14	>99%
12	Orn216	NPVEHPFGGGN<u>H</u>QHIGKPST	$C_{91}H_{139}N_{29}O_{28}$	2087.03	2186.23	>99%
13	1-TriaA216	NPVEHPFGGGN<u>H</u>QHIGKPST	$C_{91}H_{135}N_{31}O_{28}$	2112.01	2112.42	>99%
14	Gln216	NPVEHPFGGGN<u>H</u>QHIGKPST	$C_{91}H_{137}N_{29}O_{29}$	2101.01	2100.37	>99%
15	hGln216	NPVEHPFGGGN<u>H</u>QHIGKPST	$C_{92}H_{139}N_{29}O_{29}$	2115.03	2114.28	~91%
16	Arg216	NPVEHPFGGGN<u>H</u>QHIGKPST	$C_{92}H_{141}N_{31}O_{28}$	2129.06	2128.58	>99%
17	Cit216	NPVEHPFGGGN<u>H</u>QHIGKPST	$C_{92}H_{130}N_{30}O_{29}$	2130.04	2129.45	>99%
18	Asn216	NPVEHPFGGGN<u>H</u>QHIGKPST	$C_{90}H_{135}N_{29}O_{29}$	2087.00	2086.50	~94%

2. Turnover assays with MINA53 and NO66

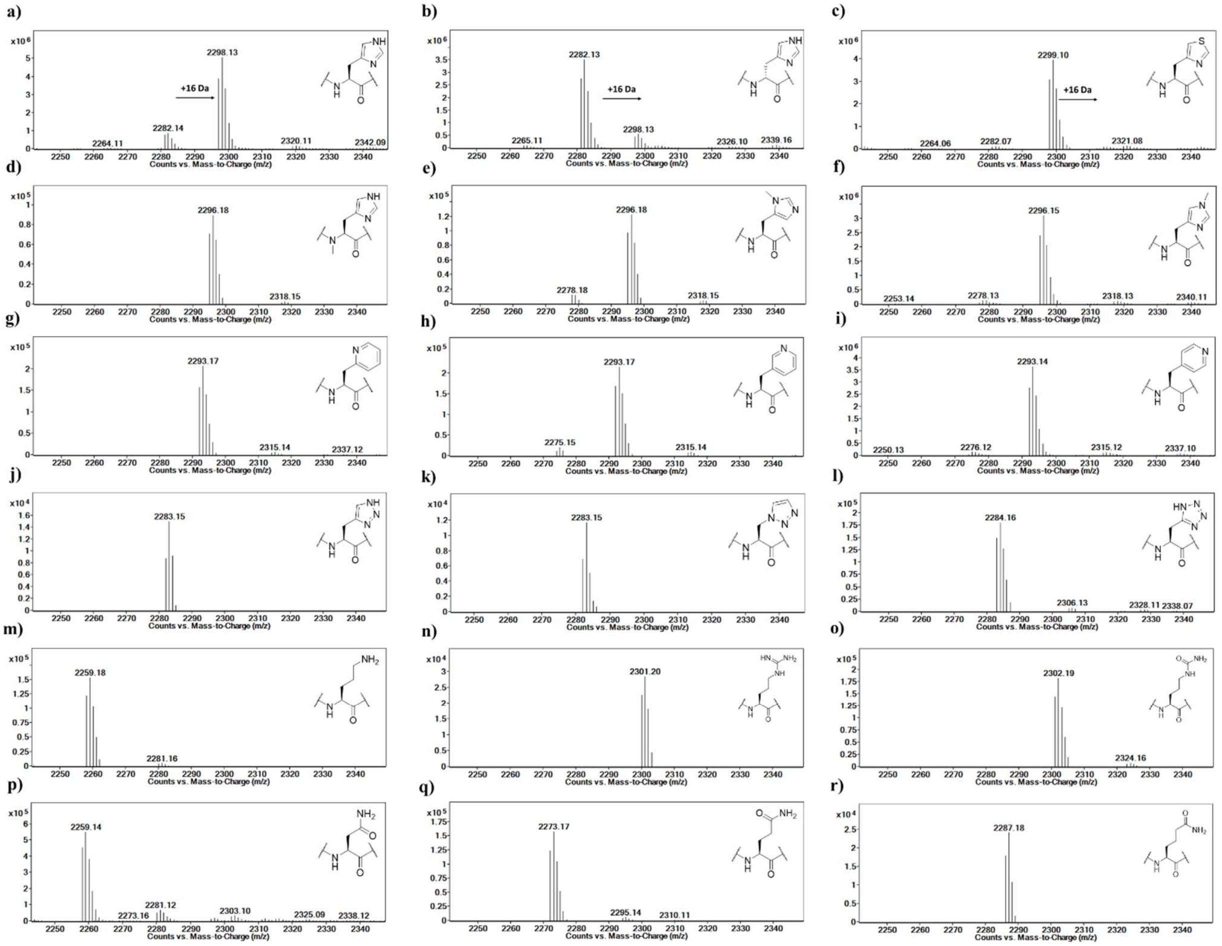

Figure S1. LC-MS data investigating the potential oxidation of Rpl27a-His39 peptides as catalysed by MINA53. a) Rpl27a-His39, b) Rpl27a-D-His39, c) Rpl27a-ThiA39, d) Rpl27a-N_{α}-His39, e) Rpl27a-N^{π}-Me-His39, f) Rpl27a-N^{τ}-me-His39, g) Rpl27a-2PyrA39, h) Rpl27a-3PyrA39, i) Rpl27a-4PyrA39, j) Rpl27a-4-TriaA39, k) Rpl27a-1-TriaA39, l) Rpl27a-TetrA39, m) Rpl27a-Orn39, n) Rpl27a-Arg39 and o) Rpl27a-Cit39, p) Rpl27a-Asn39, q) Rpl27a-Gln39 and r) Rpl27a-hGln39. Conditions: 2 μM MINA53, 10 μM Rpl peptide, 100 μM ascorbate, 10 μM ferrous ammonium sulfate, 10 μM 2OG, pH 7.5, 2 hours at room temperature.

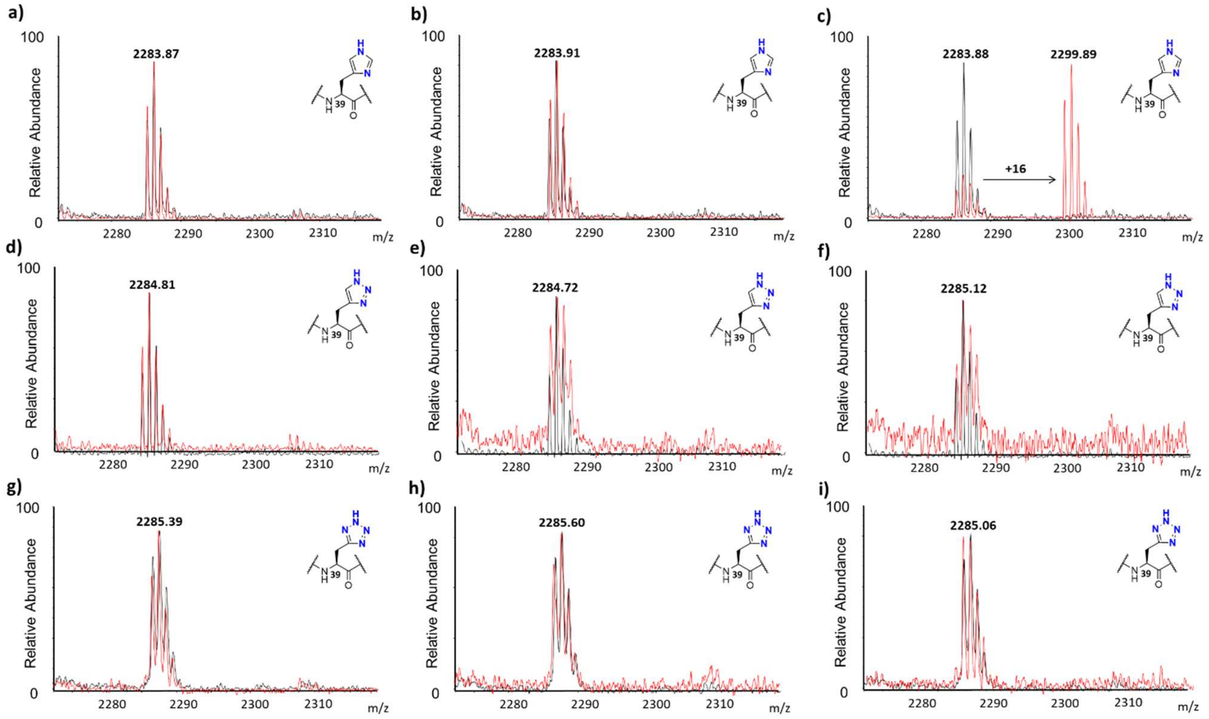

Figure S2. MALDI-TOF MS data investigating the pH dependency of Rpl27a in the presence of MINA53 in 50 mM HEPES at pH 4.5 (a, d and g), 6 (b, e and h) and 7.5 (c, f and i). a, b, c) Rpl27a-His39, d, e, f) Rpl27a-4-TriA and g, h, i) Rpl27a-TetrA. Control reactions in the absence of MINA53 are in black. Potential MINA53-catalysed reactions are in red.

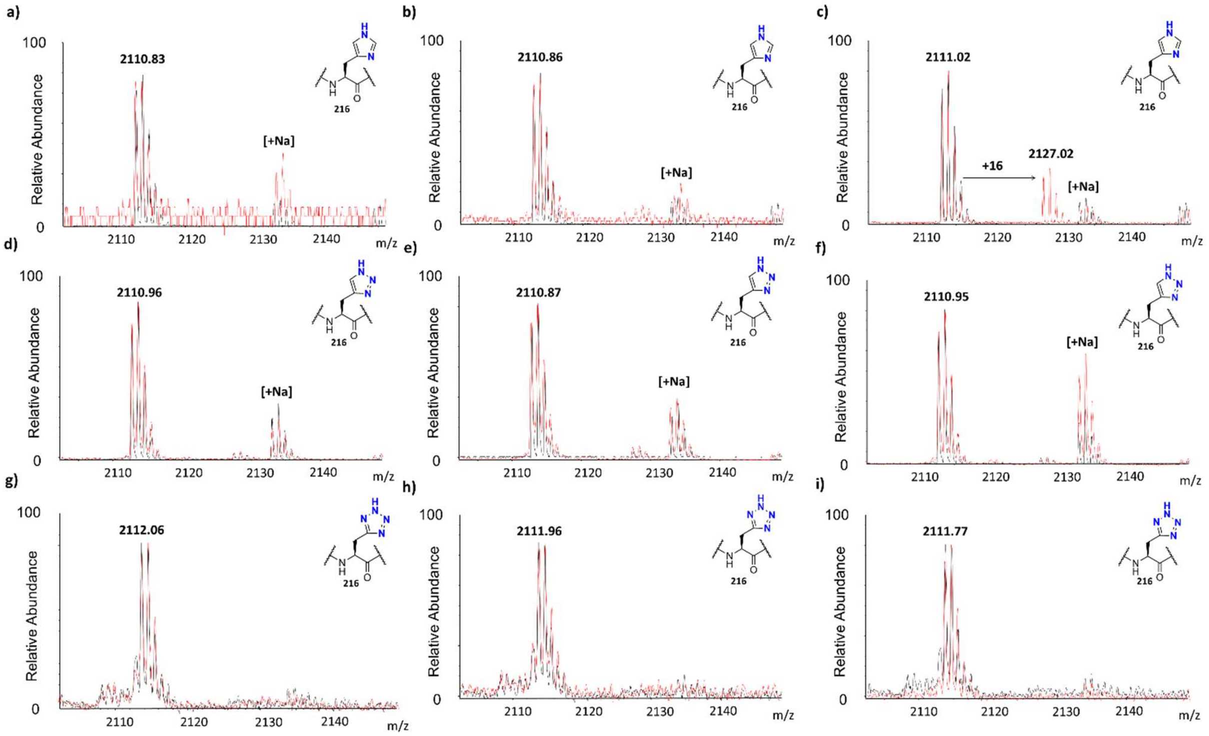

Figure S3. MALDI-TOF MS data investigating the pH dependency of Rpl8 in presence of NO66 in 50 mM HEPES at pH 4.5 (a, d and g), 6 (b, e and h) and 7.5 (c, f and i). a, b, c) Rpl8-His39, d, e, f) Rpl8-4-TriA and g, h, i) Rpl8-TetrA. Control reactions in the absence of NO66 are in black. Potential NO66-catalysed reactions are in red.

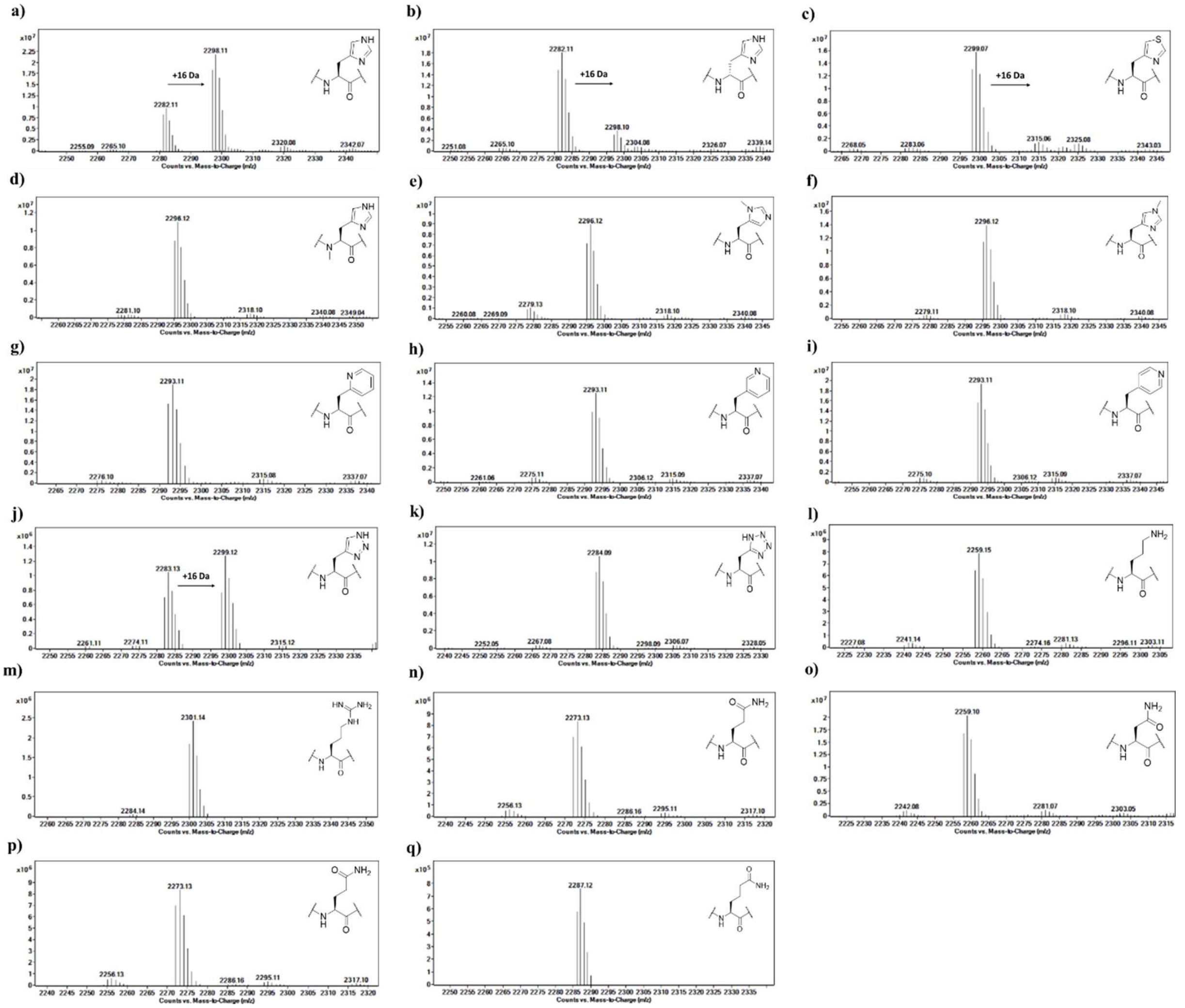

Figure S4. LC-MS data showing potential oxidation of Rpl27a-His39 peptides as catalysed by MINA53. a) Rpl27a-His39, b) Rpl27a-D-His39, c) Rpl27a-ThiA39, d) Rpl27a-N_{α}-His39, e) Rpl27a-N^{π}-Me-His39, f) Rpl27a-N^{τ}-me-His39, g) Rpl27a-2PyrA39, h) Rpl27a-3PyrA39, i) Rpl27a-4PyrA39, j) Rpl27a-4-TriaA39, k) Rpl27a-TetrA39, l) Rpl27a-Orn39, m) Rpl27a-Arg39 and n) Rpl27a-Cit39, o) Rpl27a-Asn39, p) Rpl27a-Gln39 and q) Rpl27a-hGln39. Conditions: 2 μM MINA53, 50 μM Rpl27a peptide, 100 μM ascorbate, 10 μM ferrous ammonium sulfate, 10 μM 2OG, pH 7.5, 2 hours at room temperature. All reactions were analyzed by LC-MS with 3 blank injections of water between each assay sample to minimize 'carry-over'.

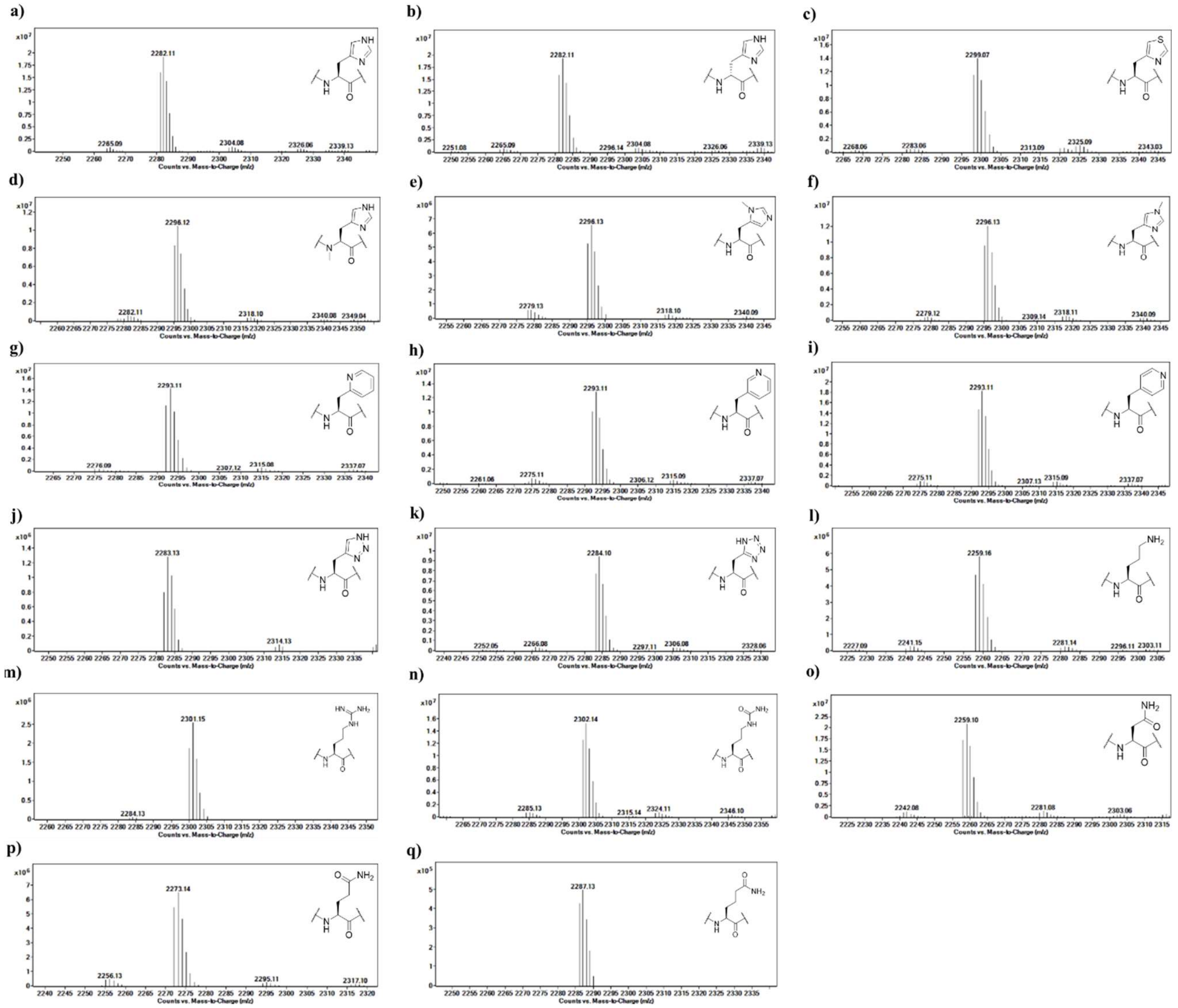

Figure S5. LC-MS data showing no-enzyme controls using Rpl27a-His39 peptides. a) Rpl27a-His39, b) Rpl27a-D-His39, c) Rpl27a-ThiA39, d) Rpl27a-N_{α}-His39, e) Rpl27a-N^{π}-Me-His39, f) Rpl27a-N^{τ}-me-His39, g) Rpl27a-2PyrA39, h) Rpl27a-3PyrA39, i) Rpl27a-4PyrA39, j) Rpl27a-4-TriaA39, k) Rpl27a-TetrA39, l) Rpl27a-Orn39, m) Rpl27a-Arg39 and n) Rpl27a-Cit39, o) Rpl27a-Asn39, p) Rpl27a-Gln39 and q) Rpl27a-hGln39. No enzyme control as enzyme reaction using 50 µM Rpl27a peptides (as in Figure S4) acidified with final concentration of 1% formic acid (v/v). All reactions were analyzed by LC-MS with 3 blank injections of water 3 between each assay sample to minimize 'carry-over'.

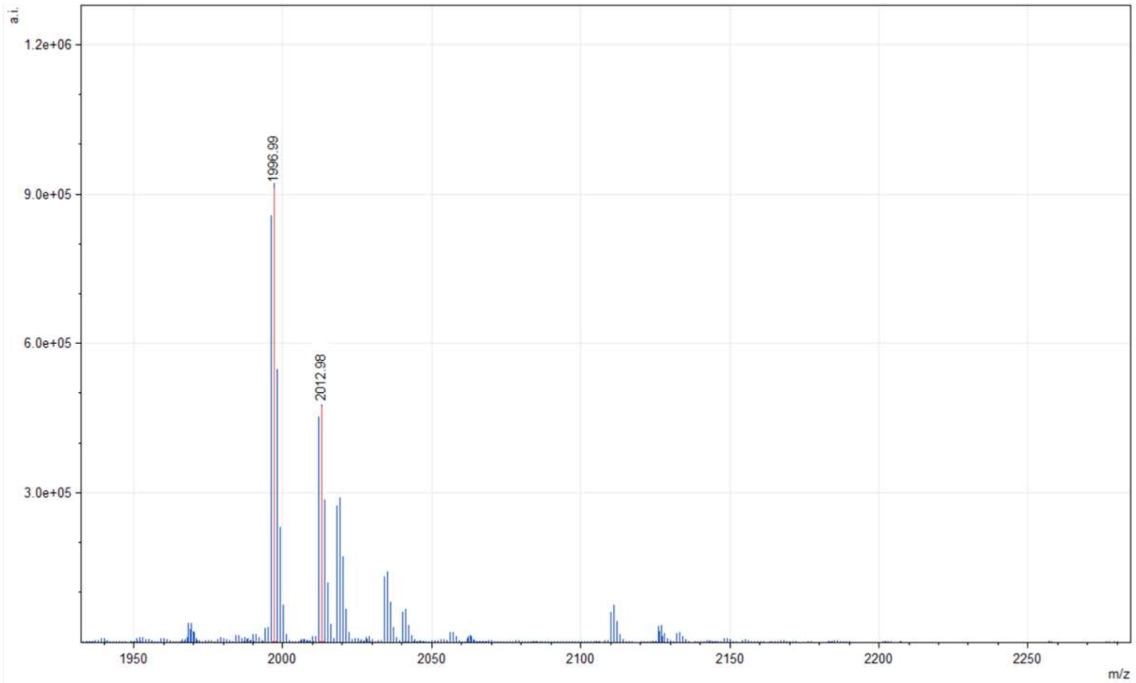

Figure S6. MALDI-TOF MS data showing evidence for hydroxylation (+16 Da) of the Rpl8-4-TriA216 peptide with NO66. This sample was used for fragmentation MALDI-TOF MS/MS analysis (Figure S8).

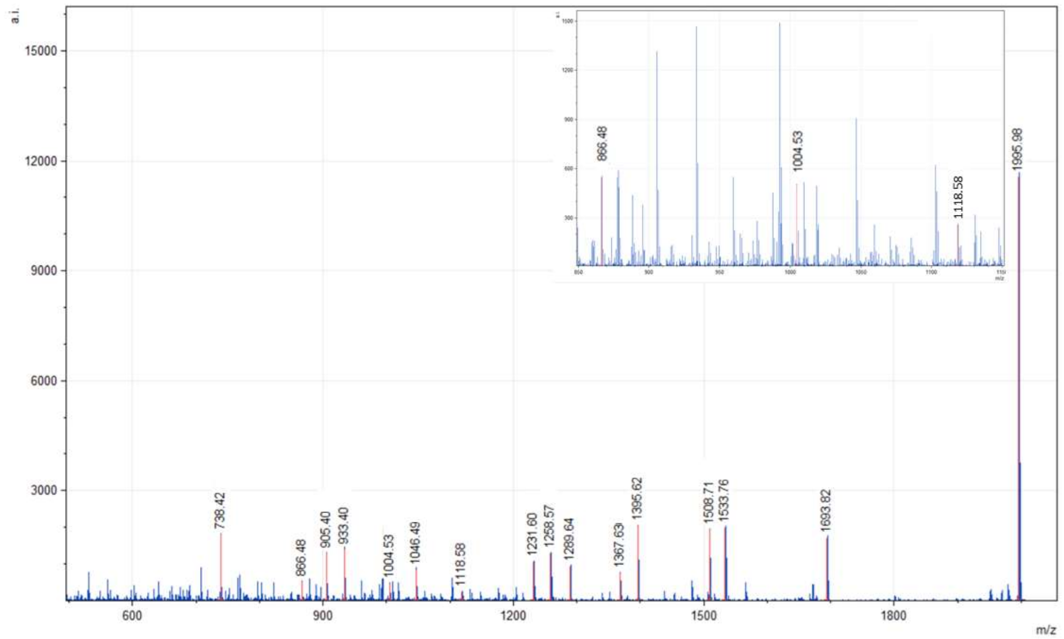

Figure S7. MALDI-TOF MS/MS fragmentation data for the unmodified Rpl8-4-TriA216 peptide. The inset shows a close up for the y_8', y_9' and y_{10}' peaks.

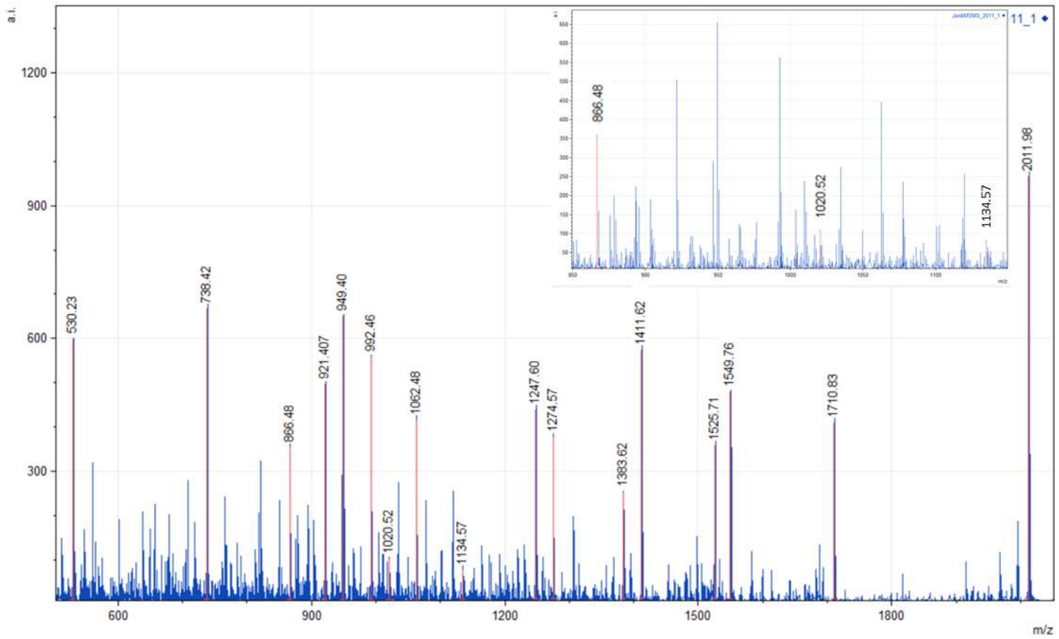

Figure S8. MALDI-TOF MS/MS fragmentation data showing the hydroxylated Rpl8-4-TriA216 peptide. The inset shows close up the y_9' peak; note the y_9' mass is 16 Da greater than the corresponding y_9' peak of the unmodified peptide (Figure S7).

3. Inhibition supporting figure

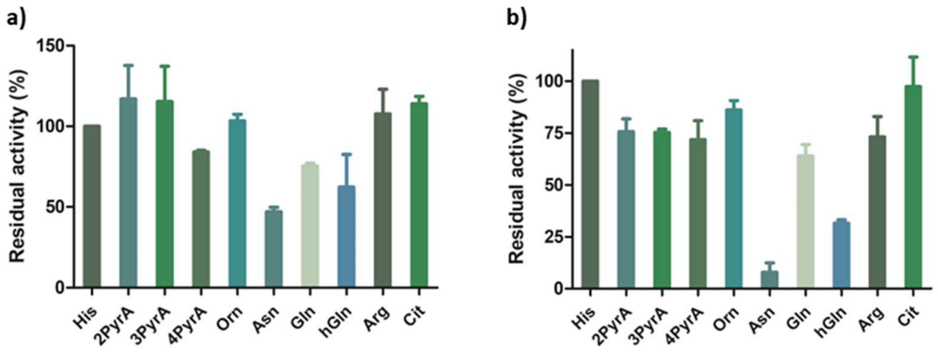

Figure S9. Results from NO66 inhibition at A) 5 μM and B) 50 μM of Rpl8-based peptides. Error bars are standard errors (SE) of duplicate analyses.

4. Molecular dynamics simulations

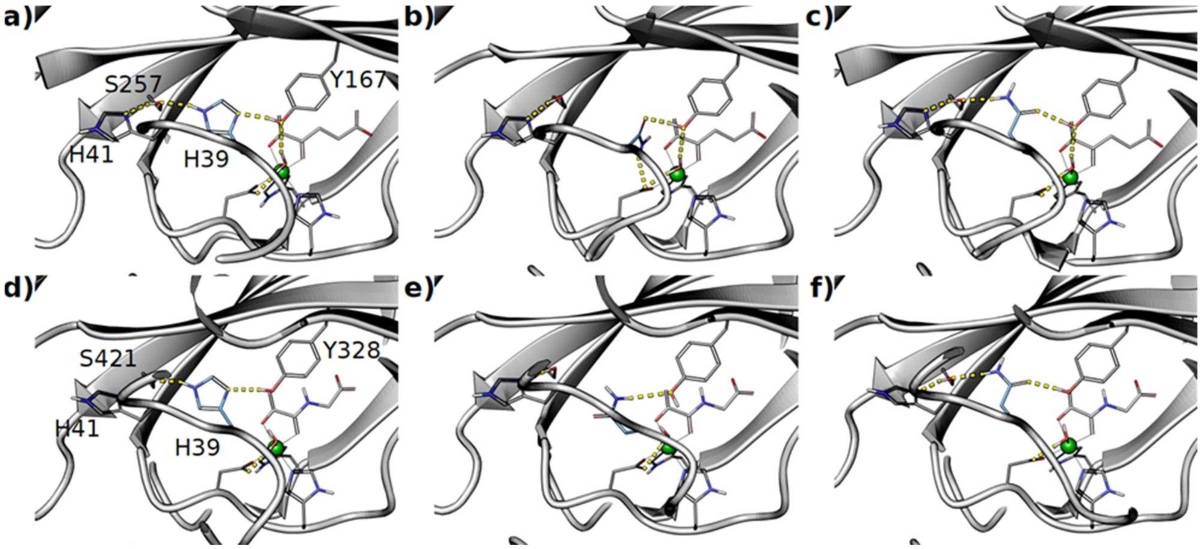

Figure S10. Average coordinates from the MD simulations of a-c): MINA53 and d-f) NO66 in complex with a,d) His39/His216, b,e) Asn39/Asn216 and c,f) Gln39/Gln216. Yellow dashed lines: hydrogen bonds. The simulations were based on crystal structures with PDB codes: ID 4BXF and 4Y3O.
